# Supplementary material for: Comparative analysis of multiple DNA double-strand break repair pathways in CRISPR-mediated endogenous tagging
Source: Commun Biol. 2025 May 13;8:749. doi: 10.1038/s42003-025-08187-5 (PMC12075812; doi:10.1038/s42003-025-08187-5)
Supplement: Supplementary file 2 — Supplementary Information [file 42003_2025_8187_MOESM2_ESM.pdf]

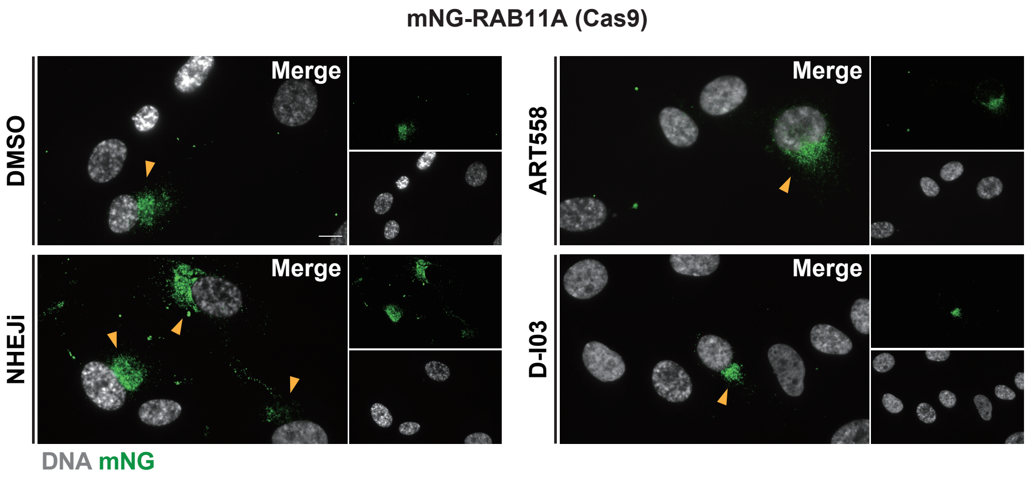

Figure S1: Related to Figure 1

Representative images from cells with Cas9-mediated mNG tagging of RAB11A. Cells were treated with the indicated inhibitors for 24 hours after electroporation, and were fixed and analyzed 6 days after electroporation. Yellow arrowheads indicate cells with endosome-like mNG signals. Scale bar: 10 µm.

a

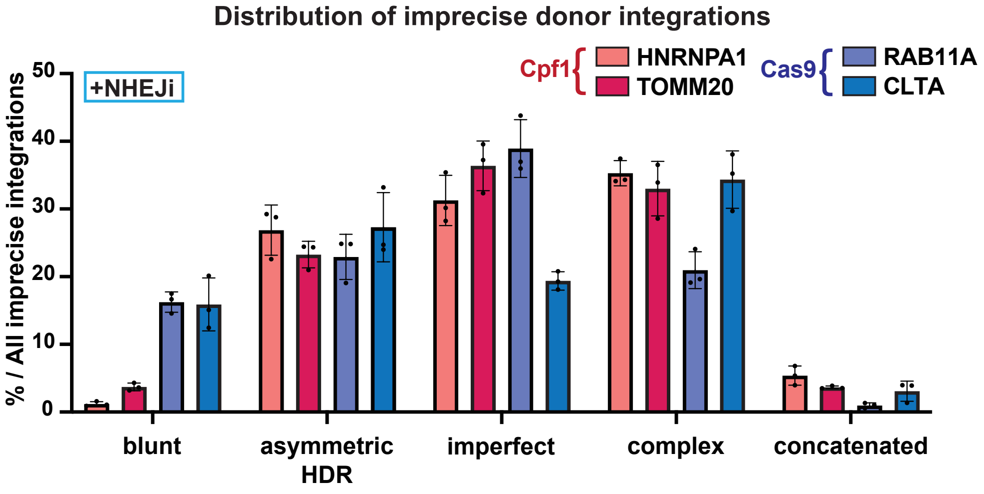

b

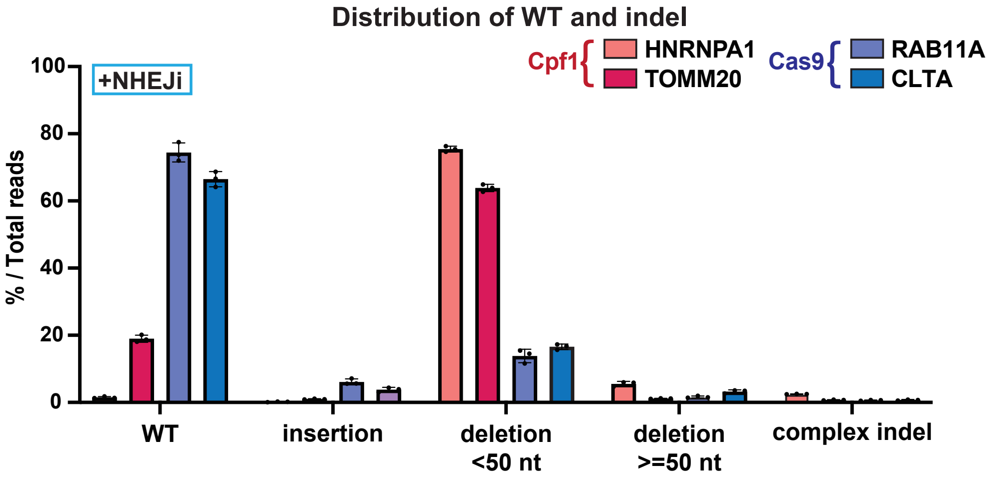

Figure S2: Related to Figure 1

a, Distribution of imprecise integration events classified by knock-knock across the targeted genes. For each category, percentage within total imprecise integration events are shown. **b**, Distribution of repair patterns without donor integration was shown across the targeted gene loci after knock-knock categorized each sequence read into a specific category of knock-in outcomes and calculated the frequency of each repair category. 122,496-211,434 reads were analyzed for each sample.

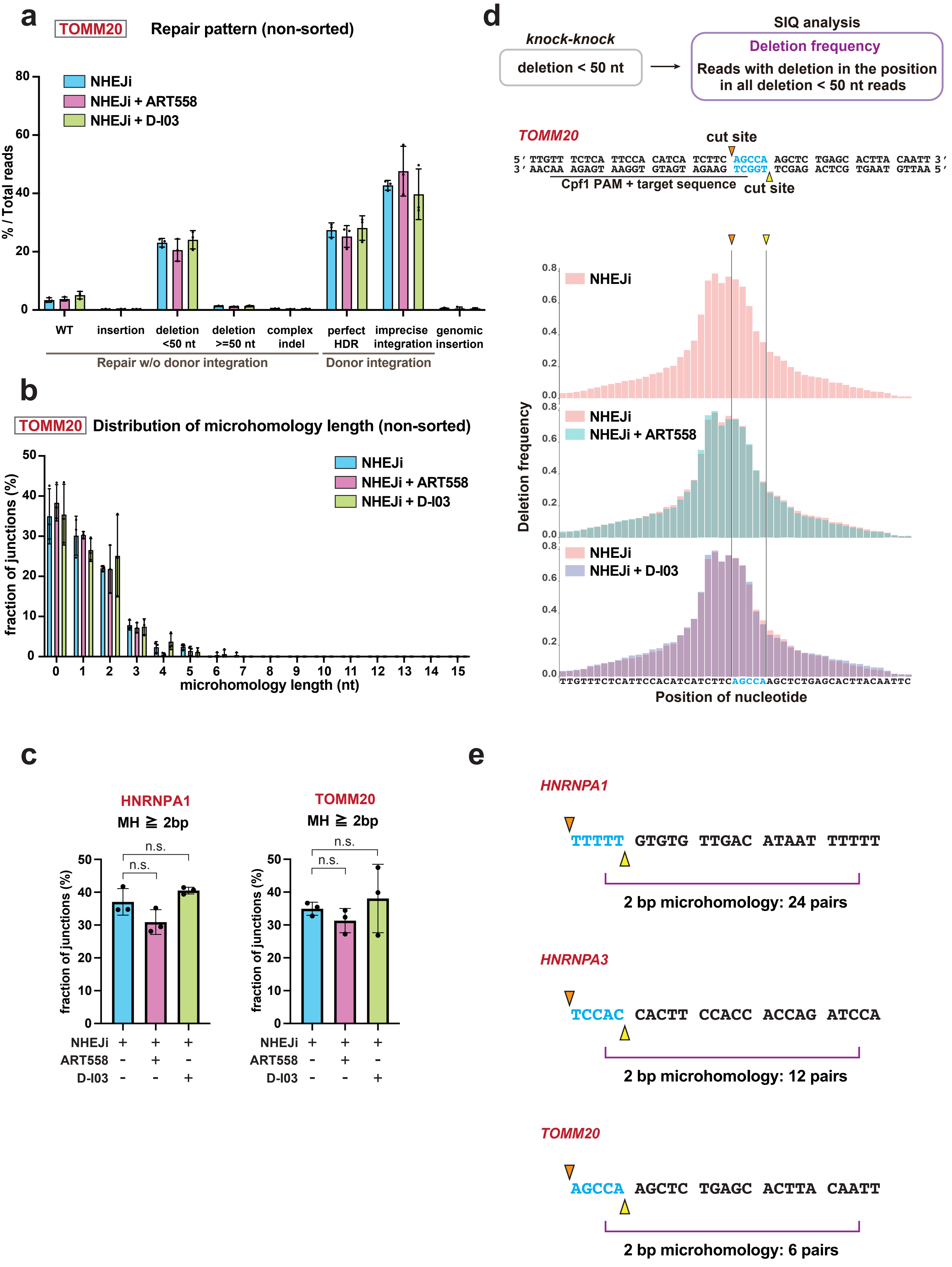
a
TOMM20
Repair pattern (non-sorted)
NHEJi
NHEJi + ART558
NHEJi + D-I03
% / Total reads
0
20
40
60
80
WT
insertion
deletion <50 nt
deletion >=50 nt
complex indel
perfect HDR
imprecise integration
genomic insertion
Repair w/o donor integration
Donor integration
b
TOMM20
Distribution of microhomology length (non-sorted)
NHEJi
NHEJi + ART558
NHEJi + D-I03
fraction of junctions (%)
0
10
20
30
40
50
0 1 2 3 4 5 6 7 8 9 10 11 12 13 14 15
microhomology length (nt)
c
HNRNPA1
MH ≧ 2bp
n.s.
n.s.
fraction of junctions (%)
0
10
20
30
40
50
NHEJi + + +
ART558 - + -
D-I03 - - +
TOMM20
MH ≧ 2bp
n.s.
n.s.
fraction of junctions (%)
0
10
20
30
40
50
NHEJi + + +
ART558 - + -
D-I03 - - +
d
knock-knock
deletion < 50 nt
SIQ analysis
Deletion frequency
Reads with deletion in the position in all deletion < 50 nt reads
TOMM20
cut site
5′ TTGTT TCTCA TTCCA CATCA TCTTC AGCCA AGCTC TGAGC ACTTA CAATT 3′
3′ AACAA AGAGT AAGGT GTAGT AGAAG TCGGT TCGAG ACTCG TGAAT GTTAA 5′
Cpf1 PAM + target sequence
cut site
NHEJi
NHEJi
NHEJi + ART558
NHEJi
NHEJi + D-I03
Deletion frequency
0.0
0.2
0.4
0.6
0.8
TTGTTTCTCATTCCACATCATCTTCAGCCAAGCTCTGAGCACTTACAATTC
Position of nucleotide
e
HNRNPA1
TTTTT GTGTG TTGAC ATAAT TTTTT
2 bp microhomology: 24 pairs
HNRNPA3
TCCAC CACTT CCACC ACCAG ATCCA
2 bp microhomology: 12 pairs
TOMM20
AGCCA AGCTC TGAGC ACTTA CAATT
2 bp microhomology: 6 pairs

Figure S3: Related to Figure 3

a, Distribution of repair patterns in Cpf1-mediated mNG tagging of TOMM20 in cells treated with the indicated inhibitors for 24 hours after electroporation. Long-read amplicon sequencing and subsequent knock-knock analysis were performed following the methodology depicted in Fig. 1e. For each sample, 10,977-19,160 sequencing reads were analyzed. **b**, Distribution of microhomology length at genome-donor junctions in Cpf1-mediated mNG tagging of TOMM20 in (a). Reads categorized as the imprecise integrations, where both sides of the donor were trimmed or one side was integrated via HDR while the other was trimmed, were analyzed. **c**, The frequency of total events of microhomology ≥2 bp at genome-donor junctions in Cpf1-mediated mNG tagging of HNRNPA1 in Figure 3a and TOMM20 in Figure S3a. Reads categorized as the imprecise integrations, where both sides of the donor were trimmed or one side was integrated via HDR while the other was trimmed, were analyzed. Data from three biological replicates are presented as mean ± S.D. and P value was calculated by a Tukey–Kramer test in this figure. n.s.: Not significant. **d**, Positional distribution of deletions within reads categorized as “deletions of less than 50 nt” by knock-knock in (a). The percentage of reads with a deletion at each nucleotide was calculated and visualized using SIQ program and SIQPlotteR. A total of 2,541-4,073 reads from three biological replicates for each condition were analyzed and plotted collectively. **e**, Schematic overview of the number of 2 bp microhomologous sequences between the Cpf1-mediated ssDNA overhang (cyan) and its 20 bp downstream sequences (black) at the *HNRNPA1*, *HNRNPA3*, and *TOMM20* loci.

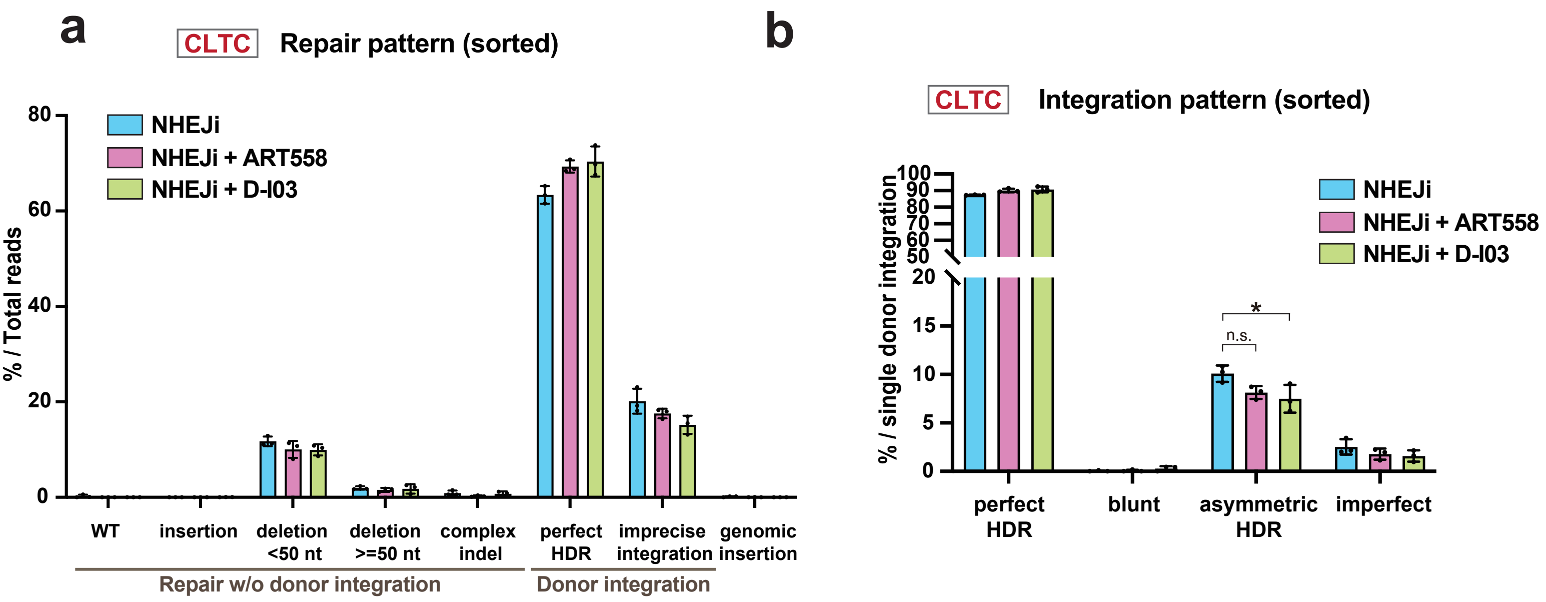

Figure S4: Related to Figure 3

a, Distribution of repair patterns in mNG tagging of CLTC within fluorescence-positive cells. Cells were knocked in, incubated, collected, and analyzed in the same manner as Fig. 3d. 59,205-125,060 reads were analyzed per sample. **b**, Distribution of donor integration patterns from knock-knock analysis of CLTC (a). For each category, the percentage within single donor DNA integration events (perfect HDR, blunt integrations, asymmetric HDR, and imperfect integrations) was calculated. Data from three biological replicates are presented as mean ± S.D. and P value was calculated by a Tukey–Kramer test in this figure. *P <0.05, n.s.: Not significant.

Supplementary Table 1: Primer sequences for PCR

For guide RNA assembly

Name	Sequence
Cpf1_crRNA_Fw	TTCTAATACGACTCACTATAGTAATTTCTACTCTTGTAGAT
HNRNPA1_crRNA_Rv	AAGGTGCTTACTTACCTAATATCTACAAGAGTAGAAATTAC
HNRNPA3_crRNA_Rv	GATATGGTAGCAGAAGGTTCATCTACAAGAGTAGAAATTAC
TOMM20_crRNA_Rv	CTGAAGATGATGTGGAATGAATCTACAAGAGTAGAAATTAC
CLTC_crRNA_Rv	TTCATCTCACATGCTGTACCATCTACAAGAGTAGAAATTAC
Cas9_Universal_sgRNA_Fw	TTCTAATACGACTCACTATAG
Cas9_Universal_sgRNA_Rv	AAAAGCACCGACTCGGTG
Cas9_crRNA_tracrRNA	GTTTTAGAGCTAGAAATAGCAAGTTAAAATAAGGCTAGTCCGTTATCAACTTGAAA AAGTGGCACCGAGTCGGTGCTTTT
RAB11A_sgRNA_Fw	TTCTAATACGACTCACTATAGGTAGTCGTACTCGTCGTCG
RAB11A_sgRNA_ Rv	TTCTAGCTCTAAAACCGACGACGAGTACGACTACC
CLTA_sgRNA_Fw	TTCTAATACGACTCACTATAGAACGGATCCAGCTCAGCCA
CLTA_sgRNA_Rv	TTCTAGCTCTAAAACTGGCTGAGCTGGATCCGTTC
HDR reporter_sgRNA_Fw	TTCTAATACGACTCACTATAGCGGTTCGGAAGCCAACACGT
HDR reporter_sgRNA_Rv	TTCTAGCTCTAAAACACGTGTTGGCTTCCGAACCGC

For HDR donor preparation

Name	Sequence
HNRNPA1-mNG/mScarlet_Fw	CACTTTGAAACTTTAAAAGAAAAATTGTACTTTTCAGGTGGCTATGGCGGTTCCAG CAGCAGCAGTAGCTATGGCAGTGGCAGAAGATTTGGAGCTGGTGCAGGTGCAG
HNRNPA1-mNG/mScarlet_Rv	ACTGCAATTATAATGTTAACTATGTTGCACTGCTCAGCTACATTAGGGTTATTGGGT TCATCAGCAATTTAAAAAATTATGTCAACACACAAAAAGGTGCTTACTTACCTAACT ACTTGTACAGCTCGTCCATGC
HNRNPA3-mNG/mScarlet_Fw	AATGTGCATACTTCTTTTAAAATACTATGTATATTTTCAGGTGGTTATGGATCTGGTG GTGGAAGTGGTGGATATGGTAGCAGAAGGTTCGGAGCTGGTGCAGGTGCAG
HNRNPA3-mNG/mScarlet_Rv	TAAAAAAATTATTACACTCAGTGAACAGTTCCACATATTCTTTTATCATCATAATAAA AATTTAAAGATACCTACCCTTTTCTGCTGTTTCTACTTGTACAGCTCGTCCATGC
TOMM20-mNG/mScarlet_Fw	TATTTTGAAGTTAGAATCCTAATTAAATGCTTATGACACTTTAAAAAATTATTTTTTTT TTCTTTCAGAGAATTGTAAGTGCTCAGAGCTTGGCTGAAGATGATGTGGAAGGAG CTGGTGCAGGTGCAG
TOMM20-mNG/mScarlet_Rv	ATATTTGCCCTTATTCCCCCAGAGCTGCTCAACTACCAAGAATTTTTAAAATATTTT TAACTGAGATTTTATTATGTTGACATTTGTTTCCTACTTGTACAGCTCGTCCATGC

mNG-RAB11A_Fw	GGCGCTCGGGTTACCCCTGCAGCGACGCCCCCTGGTCCCACAGATACCACTGCTGCTCCCGCCCTTTCGCTCCTCGGCCGCGCAATGGGCATGGTGAGCAAGGGCGAG
mScarlet-RAB11A_Fw	GGCGCTCGGGTTACCCCTGCAGCGACGCCCCCTGGTCCCACAGATACCACTGCTGCTCCCGCCCTTTCGCTCCTCGGCCGCGCAATGGGCATGGTGAGCAAGGGCGAGGC
mNG/mScarlet-RAB11A_Rv	GGGAGTGGCCCGGGTCCCCGAACGAGGACTGTGTAGAGTGCGAGAGCCCATGGCCTCACCTTTAAAGAGGTAGTCGTACTCGTCGTCGCGTGCACCAGCTCCTGCACC
mNG-CLTA_Fw	ACAGCGGTGGCTGCCGGGCGTGGTGTCGGTGGGTCGGTTGGTTTTTGTCTCACCGTTGGTGTCCGTGCCGTTCAGTTGCCCGCCATGGCTATGGTGAGCAAGGGCGAG
mScarlet-CLTA_Fw	ACAGCGGTGGCTGCCGGGCGTGGTGTCGGTGGGTCGGTTGGTTTTTGTCTCACCGTTGGTGTCCGTGCCGTTCAGTTGCCCGCCATGGCTATGGTGAGCAAGGGCGAGGC
mNG/mScarlet-CLTA_Rv	AGCCGGGTCTTCTTCGCCGGCGCCGGCCACTCCGTTCCCCAGCGCGGGACCGCCAGGGGCGCCGGCAGGGGCGCCGAACGGATCCAGCTCTGCACCAGCTCCTGCACC
mNG-CLTA_2nd_Fw	ACAGCGGTGGCTGCCGGG
mNG-CLTA_2nd_Rv	AGCCGGGTCTTCTTCGCCGG
CLTC-mNG_Fw	AGTGTTGCCGTCCCTCCCCAGGCACCTTTTGGTTATGGTTATACCGCACCACCGTATGGACAGCCACAGCCTGGCTTTGGGTACAGCATGGGAGCTGGTGCAGGTGCAG
CLTC-mNG_Rv	GTTGCCTGTTTTCCCCCATTATAAACTGAGAAGTGGGTAAAGACGATGTTTCAGTACGAAAATAGGTGACTACAGGATCAGCGCTTCATCCTACTTGTACAGCTCGTCCATGC
The HDR reporter donor_Fw	CCGCCATGGTAGATGGCTCC
The HDR reporter donor_Rv	CCGCTCGGTGGACGCTTC
HDR reporter cassette knock-in_Fw	CTTGTAGTAGGGCCATTTTTAAATGGCCAGACACTTGAATTTAACTTTTATTATCCCAAATATGAAAACATTACTGTTGGCACTTTGAAACTTTAAAAGAAAAATTGTACTTTTCAGGTGGCTATGGCGGTTCCAGCAG
HDR reporter cassette knock-in_Rv	AGACTCAAGGCTACAATCCAATATCAAGTTTGTTTCCCACAAATTTTGCTGATCTGAATATTAACTTTATATCCACAATTACTGCAATTATAATGTTAACTATGTTGCACTGCTCAGCTACATTAGGGTTATTGGGTTCATCAGC
HNRNPA1-HaloTag_Fw	CACTTTGAAACTTTAAAAGAAAAATTGTACTTTTCAGGTGGCTATGGCGGTTCCAGCAGCAGCAGTAGCTATGGCAGTGGCAGAAGATTTGGAGCTGGTGCAGGTGCAG
HNRNPA1-HaloTag_Rv	ACTGCAATTATAATGTTAACTATGTTGCACTGCTCAGCTACATTAGGGTTATTGGGTTCATCAGCAATTTAAAAAATTATGTCAACACACAAAAAGTTGCTTACTTACCTAACTAGCCGGAAATCTCGAGCG

For long-read amplicon sequencing

Name	Sequence
SMRT_1st_Fw_HNRNPA1	[AmC6]GCAGTCGAACATGTAGCTGACTCAGGTCACCAGGCCTTCAGCCGTTACAC
SMRT_1st_Rv_HNRNPA1	[AmC6]TGGATCACTTGTGCAAGCATCACATCGTAGCCCAACCAGAACCCAGTCAAACT
SMRT_1st_Fw_TOMM20	[AmC6]GCAGTCGAACATGTAGCTGACTCAGGTCACTGATCTGCCTCCTTTGTTAACTTG
SMRT_1st_Rv_TOMM20	[AmC6]TGGATCACTTGTGCAAGCATCACATCGTAGCTAGCGAAGCTCACAAGGCT
SMRT_1st_Fw_RAB11A	[AmC6]GCAGTCGAACATGTAGCTGACTCAGGTCACGCAGTGAAGAAGCTCATTAAGACAAC
SMRT_1st_Rv_RAB11A	[AmC6]TGGATCACTTGTGCAAGCATCACATCGTAGGAAGGTAGAGAGAGTTGCCAAATGG
SMRT_1st_Fw_CLTA	[AmC6]GCAGTCGAACATGTAGCTGACTCAGGTCACAGCCATGTAGCTATTAACATCTCCCTG
SMRT_1st_Rv_CLTA	[AmC6]TGGATCACTTGTGCAAGCATCACATCGTAGCCAACACTCTGTACACCTTAAGTGC
SMRT_1st_Fw_CLTC	[AmC6]GCAGTCGAACATGTAGCTGACTCAGGTCACTGGCTTGCCTTCAGGGTGTTTTC
SMRT_1st_Rv_CLTC	[AmC6]TGGATCACTTGTGCAAGCATCACATCGTAGGCCTCCCTAATGCCTCAGTATCCA

Supplementary Table 2: Target site sequences of guide RNA

Target gene	Cas nuclease	Target sequence
HNRNPA1 (Human)	Cpf1	ATTAGGTAAGTAAGCACCTT
HNRNPA3 (Human)	Cpf1	GAACCTTCTGCTACCATATC
TOMM20 (Human)	Cpf1	TCATTCCACATCATCTTCAG
CLTC (Human)	Cpf1	GGTACAGCATGTGAGATGAA
RAB11A (Human)	Cas9	GGTAGTCGTACTCGTCGTCG
CLTA (Human)	Cas9	GAACGGATCCAGCTCAGCCA
Adenylate kinase 2 (Mouse)	Cas9	CGGTTCGGAAGCCAACACGT

Supplementary Table 3: The information about the sequencing results

Run Name	Reagents	Sample Name	Barcode	No. of HiFi reads (>=QV40)	Total Bases (bp)	DeepConsensus	Figure
amplicon_241008_spk3bc_20241011_run01	Binding Kit 3.2<br>Sequencing Kit 2.0<br>Sequencing Primer V3.2	HNRNPA1_DMSO-1	BC1081	97,292	257,141,146	v1.2	1
		HNRNPA1_DMSO-2	BC1082	188,243	500,904,523		1
		HNRNPA1_DMSO-3	BC1083	215,279	574,667,546		1
		HNRNPA1_NHEJi-1	BC1084	279,029	810,691,025		1
		HNRNPA1_NHEJi-2	BC1085	319,727	933,194,847		1
		HNRNPA1_NHEJi-3	BC1086	151,916	435,457,013		1
		HNRNPA1_ART558-1	BC1087	240,848	648,000,342		1
		HNRNPA1_ART558-2	BC1088	204,085	554,975,877		1
		HNRNPA1_ART558-3	BC1089	227,940	615,070,404		1
		HNRNPA1_D-I03-1	BC1090	150,942	406,430,873		1
		HNRNPA1_D-I03-2	BC1091	256,246	679,193,624		1
		HNRNPA1_D-I03-3	BC1092	261,526	703,817,613		1
amplicon_230510_spk3bc_20230517_run01	Binding Kit3.1/Sequencing Kit2.0	HNRNPA1-1	BC1072	209,322	549,828,648	v1.2	1, S2
		HNRNPA1-2	BC1073	122,496	324,759,844		1, S2
		HNRNPA1-3	BC1074	171,461	452,522,572		1, S2
		TOMM20-1	BC1075	131,724	147,605,594		1, S2
		TOMM20-2	BC1076	211,434	241,442,654		1, S2
		TOMM20-3	BC1077	184,362	207,379,789		1, S2
		RAB11A-1	BC1084	203,958	311,548,040		1, S2
		RAB11A-2	BC1079	175,255	190,005,966		1, S2
		RAB11A-3	BC1080	124,304	311,548,040		1, S2
		CLTA-1	BC1081	146,635	279,196,586		1, S2
		CLTA-2	BC1082	205,758	388,830,301		1, S2
		CLTA-3	BC1083	165,855	315,453,488		1, S2
amplicon_230602_spk3bc_20230606_run01	Binding Kit3.2/Sequencing Kit2.0	HNRNPA1_NHEJi-1	BC1046	157,645	481,181,496	v1.2	3 (non-sorted)
		HNRNPA1_NHEJi-2	BC1047	145,841	453,348,742		3 (non-sorted)
		HNRNPA1_NHEJi-3	BC1048	151,343	454,731,939		3 (non-sorted)
		HNRNPA1_ART558-1	BC1049	230,164	723,083,080		3 (non-sorted)
		HNRNPA1_ART558-2	BC1050	193,847	605,100,475		3 (non-sorted)
		HNRNPA1_ART558-3	BC1064	174,940	554,503,991		3 (non-sorted)
		HNRNPA1_D-I03-1	BC1052	165,286	517,254,021		3 (non-sorted)
		HNRNPA1_D-I03-2	BC1053	166,622	518,457,349		3 (non-sorted)
		HNRNPA1_D-I03-3	BC1054	115,790	360,358,701		3 (non-sorted)
amplicon_230327_spk3bc_20230328_run01	Binding Kit3.2/Sequencing Kit2.0	HNRNPA1_NHEJi-1	BC1002	183,702	604,629,764	v1.1.0	3 (sorted)
		HNRNPA1_NHEJi-2	BC1007	157,317	519,350,435		3 (sorted)
		HNRNPA1_NHEJi-3	BC1012	226,842	746,476,738		3 (sorted)
		HNRNPA1_ART558-1	BC1003	196,337	651,542,282		3 (sorted)
		HNRNPA1_ART558-2	BC1008	163,395	545,035,511		3 (sorted)
		HNRNPA1_ART558-3	BC1013	167,824	559,259,524		3 (sorted)
		HNRNPA1_D-I03-1	BC1004	200,985	662,219,143		3 (sorted)
		HNRNPA1_D-I03-2	BC1009	183,749	604,523,605		3 (sorted)
		HNRNPA1_D-I03-3	BC1014	147,266	483,730,873		3 (sorted)
amplicon_230602_spk3bc_20230606_run01	Binding Kit 3.2<br>Sequencing Kit 2.0<br>Sequencing Primer V3.2	TOMM20_NHEJi-1	BC1055	11,854	23,465,166	v1.2	S2
		TOMM20_NHEJi-2	BC1056	15,445	29,642,695		S2
		TOMM20_NHEJi-3	BC1057	14,517	28,198,176		S2
		TOMM20_ART558-1	BC1058	15,819	35,807,111		S2
		TOMM20_ART558-2	BC1059	10,977	20,601,738		S2
		TOMM20_ART558-3	BC1060	12,948	26,018,846		S2
		TOMM20_D-I03-1	BC1061	19,160	41,421,019		S2
		TOMM20_D-I03-2	BC1065	10,390	19,277,674		S2
		TOMM20_D-I03-3	BC1063	15,251	29,064,145		S2
amplicon_231121_spk3bc_20231128_run01	Binding Kit 3.2<br>Sequencing Kit 2.0<br>Sequencing Primer V3.2	CLTC_NHEJi-1	BC1027	115,706	416,588,708	v1.2	S3
		CLTC_NHEJi-2	BC1028	116,468	426,479,325		S3
		CLTC_NHEJi-3	BC1029	59,205	216,003,640		S3
		CLTC_ART558-1	BC1030	100,200	367,151,075		S3
		CLTC_ART558-2	BC1031	121,796	448,633,004		S3
		CLTC_ART558-3	BC1032	121,999	451,680,690		S3
		CLTC_D-I03-1	BC1033	125,060	453,994,546		S3
		CLTC_D-I03-2	BC1034	84,485	308,131,976		S3
		CLTC_D-I03-3	BC1035	36,618	134,092,442		S3
			Total	8,782,430	24,360,706,000		
